# Reliability and validity of the steep ramp test to assess cardiorespiratory fitness in apparently healthy adults

**DOI:** 10.1007/s00421-025-06071-y

**Published:** 2026-01-19

**Authors:** Ingeborg A. Trul-Kreuze, Bart C. Bongers, Darcy Ummels, Caspar Mylius, Anuschka S. Niemeijer, Tim Takken, Marianne K. Nieuwenhuis, Han Houdijk, Moniek Akkerman

**Affiliations:** 1 Department of Human Movement Sciences, University of Groningen, University Medical Center Groningen, Groningen, The Netherlands; 2 Alliance of Dutch Burn Care, Burn Centre Groningen, Martini Hospital, Groningen, The Netherlands; 3 Research Group Healthy Ageing, Allied Health Care and Nursing, Hanze University of Applied Sciences, Groningen, The Netherlands; 4 Department of Nutrition and Movement Sciences, Institute of Nutrition and Translational Research in Metabolism (NUTRIM), Faculty of Health, Medicine and Life Sciences, Maastricht University, Universiteitssingel 50, 6229 ER Maastricht, The Netherlands; 5 Department of Surgery, Institute of Nutrition and Translational Research in Metabolism (NUTRIM), Faculty of Health, Medicine and Life Sciences, Maastricht University, Maastricht, The Netherlands; 6 Research Centre for Autonomy and Participation of Persons with a Chronic Illness, Academy for Speech and Language Therapy, Zuyd University of Applied Sciences, Heerlen, The Netherlands; 7 Child Development and Exercise Center, Wilhelmina Children’s Hospital, University Medical Center Utrecht, Utrecht, The Netherlands

**Keywords:** Exercise testing, Physical fitness, Healthy population, Public health, Clinimetric properties

## Abstract

**Purpose:**

To investigate the test-retest reliability and validity of the steep ramp test (SRT), a short and practical maximal exercise test on a cycle ergometer, for assessing CRF in apparently healthy adults aged 25–85 years.

**Methods:**

To determine test-retest reliability, the reliability group performed the SRT twice within 2–14 days. The intraclass correlation coefficient (ICC) for the attained work rate at peak exercise (WR_peak_) at the SRT was calculated and agreement was evaluated using Bland-Altman plots. The smallest detectable change (SDC) was calculated. To determine criterion and construct validity of the SRT, the validity group performed both the SRT and cardiopulmonary exercise testing (CPET) on a single day. Correlation analyses were used to assess the relationship between SRT WR_peak_ and oxygen uptake (V̇O_2_) at peak exercise (V̇O_2peak_), V̇O_2_ at the first ventilatory threshold (V̇O_2VT1_), and the oxygen uptake efficiency slope (OUES) achieved during CPET.

**Results:**

In the reliability group (n = 66, 35 males), the ICC for SRT WR_peak_ was 0.992. Limits of agreement ranged from − 18.6 W (-5.4%) to 24.1 W (6.5%). The SDC was 15 W, 0.2 W/kg, or 4.2%. In the validity group (n = 61, 33 males), CPET V̇O_2peak_, V̇O_2VT1_, and OUES showed Pearson’s correlation coefficients with SRT WR_peak_ of 0.936, 0.806, and 0.891, respectively. The following equation predicts V̇O_2peak_ (mL/min) from SRT performance: -280.784 + (8.680 × SRT WR_peak_ (in W)) (adjusted R^2^ = 0.874, SEE = 318.476).

**Conclusion:**

This study demonstrated excellent test-retest reliability, very high criterion validity, and high construct validity of the SRT for assessing CRF in apparently healthy adults.

**Graphical abstract:**

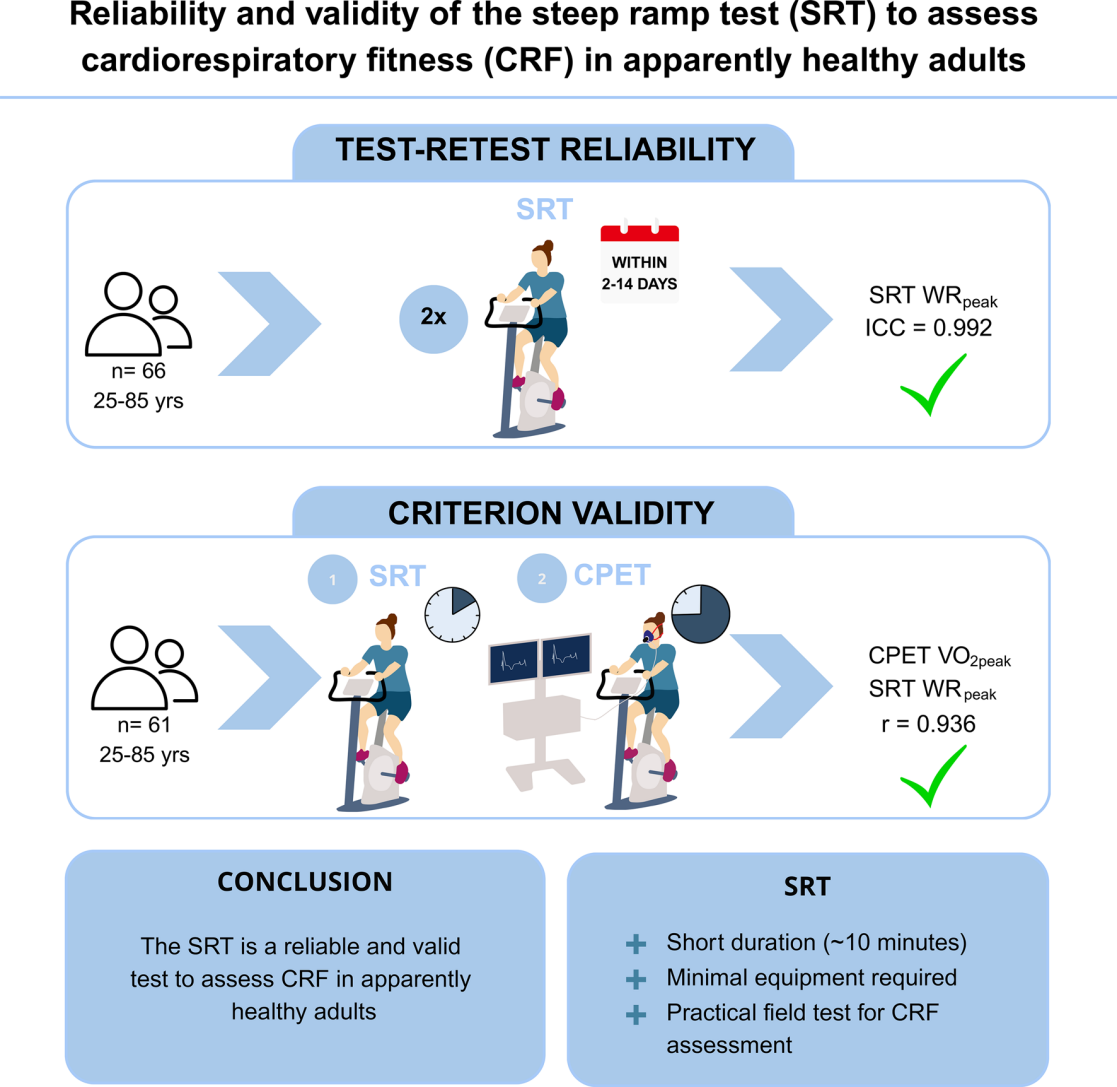

**Supplementary Information:**

The online version contains supplementary material available at 10.1007/s00421-025-06071-y.

## Introduction

Cardiorespiratory fitness (CRF) represents the integrated physiological capacity of the body to deliver and use oxygen during sustained physical activity involving large muscle groups (Caspersen et al. 1985). It is an important health indicator, as it has been consistently shown that an inverse relation exists between CRF and the risk of mortality, as well as the incidence of chronic diseases (e.g., cardiovascular disease, cancer, type 2 diabetes mellitus, dementia, depression), even in apparently healthy individuals (Han et al. 2022; Lang et al. 2024). Moreover, adding CRF to traditional risk factors like hypertension, smoking, obesity, high cholesterol, and type 2 diabetes mellitus, improves risk prediction for adverse health outcomes (Ross et al. 2016).

The strong predictive value of CRF across many health outcomes highlights its potential as a valuable tool for risk assessment that needs to be objectively measured in both public health and clinical practice (Lang et al. 2024; Ross et al. 2016). However, its use in daily practice is still limited. This might be due to a lack of awareness among healthcare professionals regarding the prognostic relevance of CRF for (long-term) health outcomes (Raghuveer et al. 2020; Ross et al. 2016). Additionally, the limited availability of resources and knowledge required to perform cardiopulmonary exercise testing (CPET), which is the gold standard for assessing CRF (American Thoracic Society; American College of Chest and Physicians 2003), may hinder its broader adoption. Submaximal alternatives, such as the Åstrand test (Åstrand and Ryhming 1954), the 6-minute walk test (6MWT) (Butland et al. 1982), or non-exercise prediction equations (Peterman et al. 2021), are available. However, these methods are prone to substantial measurement errors and/or ceiling effects, which can lead to significant inaccuracies in an individuals estimated CRF (Frost et al. 2005; Peterman et al. 2021; Raghuveer et al. 2020). Hence, there is an urgent need for a practical, yet reliable and valid alternative exercise test for CRF assessment.

The steep ramp test (SRT) is such a practical exercise test. This short maximal exercise test, performed on a cycle ergometer, involves a rapid increase in work rate that continues until voluntary exhaustion. As the achieved work rate at peak exercise (WR_peak_) is its primary outcome measure, respiratory gas analysis is not required. Currently, the SRT is used for CRF assessment in various patient populations, monitoring longitudinal changes in CRF, and personalizing interval training intensity (Trul-Kreuze et al. 2024). Its test-retest reliability has been shown to be excellent in several patient populations (Backer et al. 2007; Chura et al. 2012; Rozenberg et al. 2015), and in healthy children and adolescents (Bongers et al. 2013), with intraclass correlation values for the attained WR_peak_ ranging from 0.908 to 0.996. Its criterion validity is affirmed by strong correlations (*r* = 0.771–0.958) between oxygen uptake at peak exercise (V̇O_2peak_) measured during CPET and WR_peak_ achieved on the SRT, as reported in various patient populations (Backer et al. 2007; Bongers et al. 2015; Braam et al. 2015; Demers et al. 2024; Rozenberg et al. 2015; Weemaes et al. 2021), as well as in healthy children and adolescents (Bongers et al. 2013). In healthy adults, including older adults, the clinimetric properties of the SRT have yet to be established. Therefore, this study aimed to investigate the test-retest reliability, as well as the criterion and construct validity of the SRT for assessing CRF in apparently healthy adults aged 25 to 85 years. The aforementioned findings led to the hypothesis that the SRT is highly reliable and valid for assessing CRF in this population. If the SRT was found to be valid, a secondary aim of the study was to develop an equation to predict CPET V̇O_2peak_ from SRT WR_peak_.

## Materials and methods

### Participants

The data described in this cross-sectional study were obtained as part of the LIfelong Fitness Testing (LIFT) project, which aimed to expand the existing norm values for SRT performance in children (Bongers et al. 2013), adolescents (Bongers et al. 2013), and young adults (Werkman et al. 2020) with age- and sex-specific norm values for adults aged 25–85 years, as well as to investigate the clinimetric properties and physiological burden of the SRT. Eligible for this study were individuals aged 25–85 years without evident health conditions. In line with the aims of the LIFT project, individuals who did not comply with the Dutch physical activity guidelines, which recommend at least 2.5 h of moderate-to-intense physical activity per week (Weggemans et al. 2018), were excluded. Moreover, individuals who performed sports activities for > 10 h per week were also excluded (McKinney et al. 2019). Additional exclusion criteria were a body mass index (BMI) ≥ 30 kg/m^2^, current pregnancy, and/or absolute contra-indications for maximal exercise testing. In case of relative contraindications, a physician was consulted to decide whether a participant could safely participate.

For the current study, two participant groups were formed: one for assessing reliability and one for evaluating the validity of the SRT. The goal was to include at least 60 participants in each group, with an equal distribution of 5 males and 5 females in each 10-year age range within both groups. Given an expected intraclass correlation coefficient (ICC) of 0.90 or higher, a precision of 0.05, and a confidence level of 95%, a sample size of at least 57 participants was found to be required (Arfin 2024) for the reliability group. This sample size was also considered sufficient for the validity group based on the COnsensus-based Standards for the selection of health Measurement Instruments (COSMIN) study design checklist (Mokkink et al. 2019).

The study protocol was approved by the Medical research Ethics Committees United (MEC-U; registration number R21.088). Written informed consent was obtained from all participants before study enrolment. Reporting of the data was conducted according to the COSMIN Reporting Guideline (Gagnier et al. 2021).

### Procedures and measures

Individuals were recruited to participate in either the reliability or validity group. Individuals who agreed to participate, completed several general questions regarding their age, sex, native country, smoking behavior, body height, and body mass. They also filled out three questionnaires: (1) the physical activity readiness questionnaire (PAR-Q) (Warburton et al. 2011) to check for absolute contraindications for maximal exercise testing, (2) the short questionnaire to assess health-enhancing physical activity (SQUASH) (Wendel-Vos 2003) to establish their physical activity levels, and (3) the six-dimensional EuroQol questionnaire (EQ-6D) (Hoeymans et al. 2005) to assess their self-reported health status. Participants who met the inclusion criteria, were invited for on-site measurements. Reliability measurements were conducted at Zuyd University of Applied Sciences (Heerlen, the Netherlands), Sportsvision fitness center (Gasteren, the Netherlands), and the Martini hospital (Groningen, the Netherlands). Validity measurements were conducted at Hanze University of Applied Sciences (Groningen, the Netherlands). All measurements were performed by trained assessors. Details regarding the used materials can be found in Supplemental Information 1.

#### Anthropometry

Anthropometric measurements were performed prior to the first exercise test. Body height was measured using a stadiometer with 0.5 cm accuracy (Supplemental Information 1). Body mass was assessed with a weighing scale with 0.1 kg accuracy (Supplemental Information 1). Participants wore light clothing but no shoes during these measurements. Body mass index (kg/m^2^) was calculated as the ratio of body mass (kg) and body height (m) squared. Hip and waist circumference were measured using measuring tape to the nearest 0.1 cm (Supplemental Information 1). Percent body fat and subsequent fat-free mass (FFM) were determined three times at the right side of the body using a skinfold caliper measuring subcutaneous fat at the biceps, triceps, subscapular, and supra-iliac regions (Supplemental Information 1). The average of the first two measurements of the skinfolds was used to calculate skinfold thickness. In case the first and the second measurement differed > 10%, the average of all three measurements was used (Vereniging voor Sportgeneeskunde 2017). Skinfold thickness was used to estimate the percentage of body fat, using the standard equations as described by Durnin and Womersley (1974). The percentage body fat was then used to calculate FFM (kg).

#### Procedures test-retest reliability testing

To evaluate the test-retest reliability of the SRT, participants in the reliability group performed the SRT twice with a 2- to 14-day interval between tests. Prior to the second test, participants were asked whether there had been any changes in their health status (e.g., acute illnesses) since the first test. In apparently healthy individuals who do not undergo an intervention, no meaningful change in cardiorespiratory fitness is expected over a period of 2 weeks. Participants were blinded to test results, and the assessors did not review the previous test results before administering the second SRT.

##### Steep ramp test

The SRT was executed on a calibrated electronically-braked cycle ergometer (Supplemental Information 1), with the SRT pre-programmed in the corresponding software. Seat height was adjusted to the participant’s leg length, handlebar position was adjusted for comfort, and foot straps were securely fastened to minimize the risk of slipping off the pedals. Heart rate was monitored using a heart rate belt worn around the chest (Supplemental Information 1). Before and immediately after the test, participants were asked to rate their perceived exertion using the 6–20 Borg-scale (Borg 1982). Each participant received detailed instructions regarding the standardized test protocol, emphasizing the importance of delivering a maximal effort.

The test started with a 1-minute resting period, during which resting heart rate was recorded, while the participant sat in upright position on the cycle ergometer. This was followed by a 3-minute warm-up phase of unloaded pedaling at a frequency between 70 and 80 rpm. Subsequently, the work rate was increased by 25 W every 10 s, in a ramp-like manner. Participants were instructed to remain seated during the test while maintaining a constant pedaling frequency between 70 and 80 rpm, and at least above 60 rpm, until voluntary exhaustion. Work rate (WR), heart rate (HR), and pedaling frequency (rpm) were continuously monitored during the test. The test was terminated when participants could no longer maintain a pedaling frequency above 60 rpm, despite strong verbal encouragement. After the pedaling frequency dropped below 60 rpm, the work rate was adjusted to 25 W, or slightly higher or lower if preferred by the participant, for a 3-minute active cooling-down phase.

SRT performance was considered valid when participants met subjective criteria of maximal effort (e.g., unsteady biking, sweating, facial flushing, hyperpnea). The attained WR_peak_ was defined as the highest work rate attained during the test, at the point at which the participant could no longer maintain a pedaling frequency > 60 rpm. Heart rate at peak exercise (HR_peak_) was defined as the corresponding heart rate at WR_peak_.

#### Procedures criterion and construct validity testing

To establish the criterion and construct validity of the SRT for CRF assessment, participants in the validity group performed the SRT followed by CPET. For practical reasons, both tests were scheduled on a single day, with at least 30 min of rest in between. The sequence of the tests was not randomized. Participants first performed the SRT, followed by CPET, because the latter test was expected to induce more physiological burden compared to the SRT (Trul-Kreuze et al. 2024), and therefore takes longer to recover from.

During both the SRT and CPET, participants wore a facemask for respiratory gas analysis. The facemask was connected to a breath-by-breath metabolic cart (Supplemental Information 1) that was calibrated for respiratory gas analysis measurements (ambient air and a gas mixture of 5% carbon dioxide and 15% oxygen) and volume measurements (3-L syringe) according to the manufacturer’s instructions. This metabolic cart exhibited a measurement error of 2.85 ± 2.22% (Van Hooren et al. 2024). Oxygen uptake (V̇O_2_), carbon dioxide production, and minute ventilation (V̇E) data were averaged at 10 s intervals and saved at the corresponding software program.

##### Steep ramp test

The SRT protocol was identical to the protocol used in the reliability group, with the addition of continuous respiratory gas analysis and oxygen saturation measurements during the test. For safety reasons, blood pressure was measured before and immediately after the test.

##### Cardiopulmonary exercise testing

Cardiopulmonary exercise testing was performed using a calibrated electronically-braked cycle ergometer, with the protocol pre-programmed in the corresponding software (Supplemental Information 1). Seat height was adjusted to the participant’s leg length, handlebar position was adjusted for comfort, and foot straps were securely fastened to minimize the risk of slipping off the pedals. Heart rate was monitored continuously using 12-lead electrocardiography (ECG) during the rest phase, the testing phase, and the cooling-down phase. Oxygen saturation was measured continuously, and blood pressure was measured before, directly after, and every 3 min during the testing phase for safety reasons. Before and immediately after the test, participants were asked to rate their perceived exertion using the 6–20 Borg-scale (Borg 1982). Each participant received detailed instructions regarding the standardized test protocol, emphasizing the importance of performing a maximal effort.

The test started with a 1-minute resting period, during which resting heart rate was recorded and the ECG was assessed for deviations, while the participant sat in upright position on the cycle ergometer. This was followed by a 3-minute warm-up phase of unloaded pedaling at a frequency between 70 and 80 rpm. After that, the work rate increased automatically, with gradually increments ranging between 5 and 35 W/min, depending on the participant’s estimated WR_peak_ based on age, sex, body weight and activity level, aiming to reach maximal effort within 8–12 min. Participants were instructed to remain seated during the test while maintaining a constant pedaling frequency between 70 and 80 rpm, and at least above 60 rpm, until voluntary exhaustion. The test was terminated when participants could no longer maintain the pedaling frequency above 60 rpm, despite strong verbal encouragement, or when abnormalities in ECG, blood pressure, or oxygen saturation occurred. After the testing phase, work rate was set to 25 W, or slightly higher or lower if preferred by the participant, for at least 3 min of active cooling-down.

To check whether participants performed maximal effort, the criteria of Wagner et al. (2020) were used. Participants aged 20–39, 40–59, and ≥ 60 years, had to reach a respiratory exchange ratio at peak exercise (RER_peak_) ≥ 1.13, ≥ 1.10, and ≥ 1.06, or a HR_peak_ of ≥ 93%, ≥ 92%, and ≥ 89% of their predicted HR_peak_, respectively. V̇O_2peak_ was calculated as the average V̇O_2_ during the last 30 s prior to test termination.

 V̇O_2_ at the first ventilatory threshold (V̇O_2VT1_), which is the point during incremental exercise at which V̇E starts to increase at a faster rate than V̇O_2_ (Wasserman and McIlroy 1964), was determined by two independent observers, according to the V-slope method (Beaver et al. 1986) and the ventilatory equivalents method (Davis et al. 1980). As an objective and effort-independent indicator of CRF, the oxygen uptake efficiency slope (OUES) was derived from the linear relationship between the logarithm of V̇E and V̇O_2_ from the start of the work rate increments up to V̇O_2peak_ (Baba et al. 1996), or up to the onset of a V̇O_2_ plateau.

### Statistical analysis

Statistical analyses were performed using the Statistical Package for the Social Sciences version 25 (SPSS Inc., Chicago, IL). Statistical significance was assumed at the level of *p* ≤ 0.05. Continuous variables were assessed for normal distribution by histograms, Q-Q plots, and the Kolmogorov-Smirnov test. Patient characteristics and exercise test outcomes were presented as means ± SD or, as medians and interquartile ranges (IQR) in case of non-normal distributions.

Test-retest reliability of the SRT was evaluated using two-way random effects intraclass correlation coefficients (ICC(2,1) (Shrout and Fleiss 1979), calculated for WR_peak_, WR_peak_ normalized for body mass (W/kg), and WR_peak_ normalized for FFM (W/kg FFM) between the first and the second SRT. ICC values < 0.50 were considered to indicate poor reliability, values between 0.50 and 0.75 moderate reliability, 0.75 to 0.90 good reliability, and values > 0.90 excellent reliability (Koo and Li 2016). Paired samples *t*-tests, or Wilcoxon signed-ranks tests in case of non-normal distributions, were performed to assess the differences in SRT performance and RPE between the first and second SRT. A Bland-Altman plot was used to determine agreement for WR_peak_ between both SRTs. To check for heteroscedasticity, Pearson correlation coefficients or, in case of non-normal distributions, Spearman’s rho coefficients, were calculated between the mean values of both SRTs and the differences. Limits of agreement (LoA) were calculated for absolute WR_peak_ and WR_peak_ normalized for body mass. The smallest detectable change (SDC) was calculated as 1.96 x √2 x standard error of the mean (SEM) for absolute WR_peak_ and WR_peak_ normalized for body mass (De Vet et al. 2006) to evaluate which differences in WR_peak_ reflect true changes in SRT performance.

Criterion validity of the SRT as a measure of CRF was evaluated by calculating Pearson correlation coefficients, or Spearman’s rho in case of non-normal distributions, between the primary outcome of the SRT (WR_peak_) and the primary outcome of the gold-standard measure of CRF (CPET V̇O_2peak_). Construct validity of the SRT as a measure of CRF was evaluated by testing the hypotheses that SRT WR_peak_ would demonstrate strong correlations with CPET V̇O_2VT1_ and CPET OUES. Pearson correlations were applied to normally distributed data, and Spearman’s rho to non-normally distributed data. For both criterion and construct validity, correlations between 0 and 0.25 were considered to indicate very low validity, values between 0.26 and 0.49 low validity, values between 0.50 and 0.69 moderate validity, 0.70 to 0.89 high, and values > 0.90 very high validity (Munro 2005). Analyses were conducted on available data; participants with missing values were not included in the respective analyses.

To identify which independent variables in addition to SRT performance would best explain the variance in CPET V̇O_2peak_, multiple linear regression analyses using the enter method were performed. First, simple linear regression analyses were performed to determine which demographic and/or anthropometric variables were candidate predictors of CPET V̇O_2peak_. Based on the sample size, a maximum of 1 predictive variable per 10–15 participants was explored for its potential relationship with V̇O_2peak_ to prevent overfitting. Adjusted root mean square (R^2^) and standard error of estimate (SEE) values were evaluated for inclusion of the variable in the equation.

## Results

For reliability testing, 83 individuals agreed to participate. Based on the pre-test questionnaires, seven of these participants were excluded because of possible contra-indications for maximal exercise testing, five because they performed sports activities for > 10 h a week, one because of a BMI > 30 kg/m^2^, and one because he/she did not comply with the Dutch physical activity guidelines. Of the 69 individuals that were included, one became ill between the first and second SRT, one could not be tested twice within the required 14 days, and one withdrew informed consent. The remaining 66 participants performed the SRT twice and were thus included in the analyses.

For validity testing, 78 individuals agreed to participate. Based on the pre-test questionnaires, two participants were excluded because of possible contra-indications for maximal exercise testing, and three because of a BMI > 30 kg/m^2^. Of the 73 individuals that were included, nine could not be planned due to practical reasons, and one did not participate due to illness. The remaining 63 participants performed all measurements. In two participants, however, CPET was terminated prematurely: one due to a systolic blood pressure > 250 mm Hg and the other due to an unreadable ECG, which prevented adequate safety monitoring during the test. No adverse events occurred during the tests. Thus, a total of 61 participants completed all measurements and were thus included in the analyses. Characteristics of both groups are presented in Table 1.

**Table 1:** Participant characteristics

	Reliability group (*n* = 66)		Validity group (*n* = 61)	
Sex (male/female)	35/31		33/28	
Age categories (male/female)				
25–35 years	7/6		6/5	
35–45 years	5/5		6/4	
45–55 years	6/5		4/6	
55–65 years	7/7		7/6	
65–75 years	6/5		6/3	
75–85 years	4/3		4/4	
Age (years)	52.8 ± 17.1	[25.9–84.0]	52.8 ± 18.0	[25.0–84.1]
Body height (m)	1.76 ± 0.10	[1.50–1.96]	1.78 ± 0.95	[1.58–2.02]
Body mass (kg)	77.8 ± 13.1	[53.0–107.0]	75.9 ± 12.1	[53.5–107.5]
BMI (kg/m^2^)	24.9 ± 2.8	[19.3–29.9]	23.8 ± 2.5	[17.7–29.8]
Body fat (%)	28.7 ± 7.5	[5.4–43.3]	26.7 ± 6.3	[13.5–39.1]
Fat-free mass (kg)	55.4 ± 11.1	[35.5–83.0]	55.8 ± 10.9	[37.9–78.2]
Hip/waist ratio	0.93 ± 0.07	[0.74–1.15]	0.93 ± 0.09	[0.46–1.07]
Average total sport activities (min/week)	170.0 ± 145.5 [0–540]		252.8 ± [0–600]	
Self-declared health status (0-100)^†^	82.0 ± 9.30 [60–100]		84.3 ± 8.0 [68–100]	
Current smoking status				
Yes/no	6/60		2/59	

Values are presented as mean ± SD and [range] or as numbers

^†^Participants’ self-declared health condition was measured using the six-dimensional EuroQol questionnaire (EQ-6D), which evaluates health status on a scale from 0 to 100, with higher values indicating better perceived health

*BMI * body mass index

### Test-retest reliability of the steep ramp test

The mean interval between the first and second SRT was 8.5 days (± 2.2 days). All participants met the subjective criteria of a maximal effort on both SRTs.

For absolute SRT WR_peak_, the ICC (2,1) was 0.992 (95% CI: 0.986–0.995). For SRT WR_peak_ normalized for body mass the ICC (2,1) was 0.986 (95% CI: 0.977–0.992), whereas for SRT WR_peak_ normalized for FFM, the ICC (2,1) was 0.984 (95% CI: 0.974–0.990).

Absolute mean values for WR_peak_ were 370 ± 85 W, vs. 373 ± 88 W, and median RPE scores were 16.5 [IQR 12.0–20.0] vs. 17.2 [IQR 12.0–20.0]. Statistically significantly higher, but not clinically meaningful, values were observed in the second SRT compared with the first SRT (*p* = 0.047 and *p* = 0.002, respectively) (Table 2). WR_peak_ normalized for body mass, WR_peak_ normalized for FFM, and HR_peak_ did not differ significantly between both tests (Table 2).

**Table 2:** SRT results from the reliability group (*n* = 66)

	First SRT	Second SRT	*p*-value^†^
WR_peak_ (W)	370 ± 85	373 ± 88	0.047*
WR_peak_ normalized for body mass (W/kg)	4.8 ± 0.9	4.8 ± 0.9	0.058
WR_peak_ normalized for FFM (W/kg FFM)	6.7 ± 1.1	6.8 ± 1.2	0.059
HR_peak_ (beats/min)	158 ± 17	158 ± 17	0.808
Borg_6−20_ RPE_peak_ score	17.0 [12.0–20.0]	17.0 [12.0–20.0]	0.004*

Values for WR_peak_, WR_peak_ normalized for body mass or FFM, and HR_peak_ are presented as mean ± SD

Values for Borg_6−20_ RPE scores are presented as median and interquartile range [IQR]

*FFM* fat-free mass; *HR*_*peak*_ heart rate at peak exercise; *RPE* rate of perceived exertion at peak exercise; *SRT* steep ramp test; *WR*_*peak*_ work rate at peak exercise

*p < 0.05

^†^Paired samples *t*-tests were performed to compare test outcomes, except for Borg_6−20_ RPE score, where the Wilcoxon signed-ranks test was performed

**Fig. 1:**
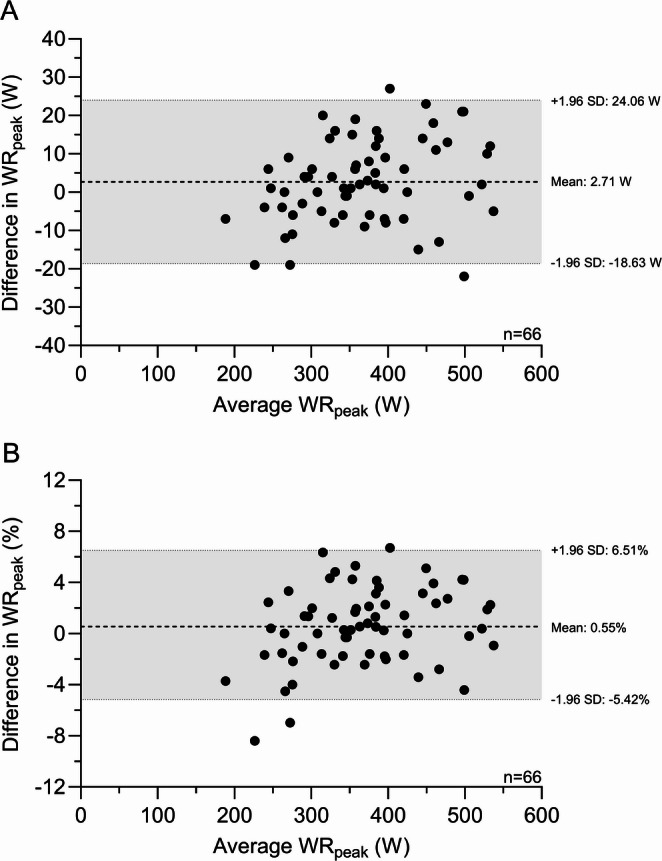
Bland-Altman plots for the WR_peak_ attained at the first and second SRT (W) (graph A) and for the WR_peak_ achieved at the first and second SRT (%) (graph B). *WR*_*peak*_ work rate at peak exercise

The Bland-Altman plot (Fig. 1) showed a mean difference of 3 ± 11 W for absolute WR_peak_ (LoA: -19 to 24 W), and of 0.03 ± 0.1 W/kg for WR_peak_ normalized for body mass (LoA: -0.3 to 0.3 W/kg). The SDC was 15 W for absolute WR_peak_ and 0.2 W/kg for WR_peak_/kg. Expressed as a percentage, the LoA ranged from − 5.4% to 6.5% (SDC 4.2%) (Fig. 1). Analysis of heteroscedasticity showed a weak but statistically significant correlation in Fig. 1 graph A (*r* = 0.25; *p* = 0.043), but not in Fig. 1 graph B (*r* = 0.215; *p* = 0.082).

### Validity of the steep ramp test

For both the SRT and CPET, all 61 participants met the criteria of a maximal effort.

#### Criterion validity

**Fig. 2:**
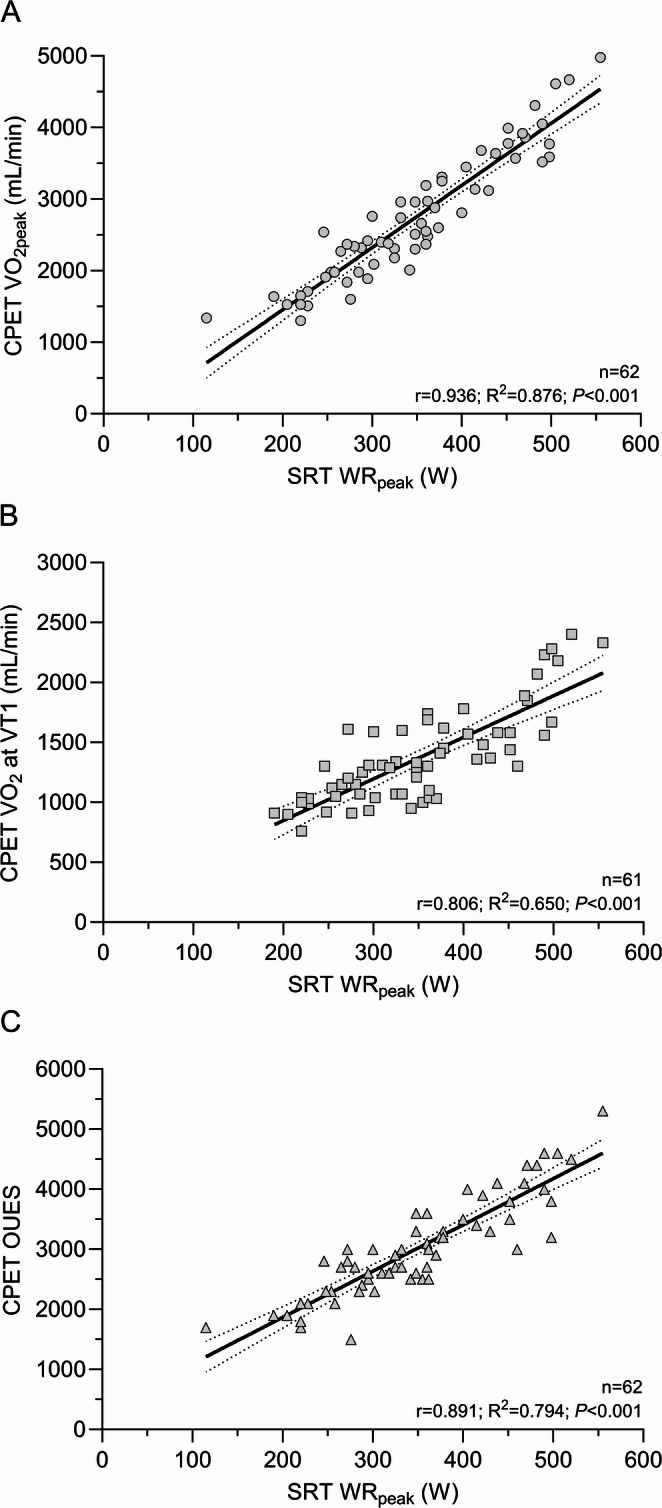
Criterion and construct validity of the SRT to assess CRF. Correlation between SRT WR_peak_ and CPET V̇O_2peak_ (graph A), between SRT WR_peak_ and CPET V̇O_2VT1_ (graph B), and between SRT WR_peak_ and CPET OUES (graph C). *CPET* cardiopulmonary exercise testing; *OUES* oxygen uptake efficiency slope; *SRT* steep ramp test; *V̇O*_2_ oxygen uptake; *V̇O*_*2peak*_ oxygen uptake at peak exercise; *VT1* first ventilatory threshold; *WR*_*peak*_ work rate at peak exercise

Test results of both the SRT and CPET are shown in Table 3. A Pearson correlation coefficient of 0.936 (*p* < 0.001) was found between SRT WR_peak_ and CPET V̇O_2peak_ (Fig. 2).

**Table 3:** SRT and CPET results from the validity group (*n* = 61)

	SRT	CPET	*p*-value^†^
Test phase duration (s)	145 [114–178]	590 [529–643]	< 0.001*
WR_peak_ (W)	349 ± 97	256 ± 90	< 0.001*
WR_peak_ normalized for body mass (W/kg)	4.6 ± 1.1	3.4 ± 1.1	< 0.001*
WR_peak_ normalized for FFM (W/kg)	6.3 ± 1.4	4.6 ± 1.3	< 0.001*
HR_peak_ (beats min)	152 ± 19.7^‡^	168 ± 17	< 0.001*
V̇O_2peak_ (mL/min)	2487 ± 924	2747 ± 897	0.001*
V̇O_2peak_ (mL/kg/min)	32.7 ± 10.7	36.5 ± 10.4	< 0.001*
V̇O_2peak_ normalized for FFM (mL/FFM/min)	44.8 ± 14.7	49.2 ± 12.3	0.002*
V̇O_2VT1_ (mL/min)	n.a.	1378 ± 399^§^	n.a.
V̇O_2VT1_ (mL/kg/min)	n.a.	18.6 ± 6.0^§^	n.a.
OUES	n.a.	3015 ± 836	n.a.
OUES/kg	n.a.	39.8 ± 9.2	n.a.
Borg_6−20_ RPE_peak_ score	17.0 [15.0–18.0]	18.0 [ 16.0–19.0]	< 0.001*

Values for WR_peak_, WR_peak_ normalized for body mass or fat-free mass, HR_peak_, V̇O_2peak_, V̇O_2peak_ normalized for body mass, V̇O_2VT1_ , and OUES are presented as mean ± SD

Values of duration of the testing phase and Borg_6−20_ RPE_peak_ scores are presented as median and interquartile range [IQR]

*CPET* cardiopulmonary exercise testing; *FFM* fat-free mass; *HR*_*peak*_ heart rate at peak exercise; *OUES* oxygen uptake efficiency slope; *RPE* rate of perceived exertion; *SRT* steep ramp test; *V̇O*_2_ oxygen uptake; *V̇O2*_*peak*_ oxygen uptake at peak exercise; VO_2*VT1*_ first ventilatory threshold; *WR*_*peak*_ work rate at peak exercise

*p < 0.05

^†^Paired samples *t*-tests were performed to compare test outcomes, except for duration of the testing phase and Borg_6–20_ RPE scores, where the Wilcoxon signed-ranks test was performed

^‡^Based on 57 participants, because HR_peak_ was not accurately measured in 4 participants

^§^Based on 60 participants, because VT1 could not be accurately identified in 1 participant

#### Construct validity

A Pearson correlation coefficient of 0.806 (*p* < 0.001) was found between SRT WR_peak_ and CPET V̇O_2VT1_ (Fig. 2), and a coefficient of 0.891 (*p* < 0.001) between SRT WR_peak_ and CPET OUES (Fig. 2).

### Predicting oxygen uptake at peak exercise from steep ramp test performance

Based on the sample size (*n* = 61) and simple linear regression analyses of the demographic and anthropometric variables, SRT WR_peak_ (W), age (years), sex (male: 0, female: 1), body height (cm), and body fat (kg) were included in the multiple linear regression analysis, as shown in Table 4. In this multiple regression model, age, sex, body height, and body fat did not contribute significantly to the prediction of CPET V̇O_2peak_ and were thus removed from the final model (Table 4). The following equation was developed to predict CPET V̇O_2peak_ from SRT WR_peak_: V̇O_2peak_ (mL/min) = -280.784 + (8.680 × SRT WR_peak_ (W)) (adjusted R^2^ = 0.874, SEE = 318.476).

**Table 4:** Linear regression analyses for predicting CPET V̇O_2peak_ (mL/min) from SRT performance (*n* = 61)

Simple linear regression				Multiple linear regression (method: enter)			
Variable		B (95% CI)	*P*-value	Variable	B (95% CI)	*P*-value	*Adjusted R* ^*2*^
V̇O_2peak_ (mL/min)							
SRT WR_peak_ (W)		**8.680 (7.830 to 9.529)**	**< 0.001**	**SRT WR**_**peak**_ **(W)**	**8.680 (7.830 to 9.529)**	**< 0.001**	**0.874**
Sex (0 = male)		**968.247 (577.046 to 1359.448)**	**< 0.001**	SRT (W) + sex (0 = male)	8.515 (7.487 to 9.543) 57.160 (-140.910 to 255.230)	< 0.001 0.566	0.873
Body height (cm)		**6150.795 (4276.353 to 8025.236)**	**< 0.001**	SRT (W) + body height (cm)	8.595 (7.416 to 9.774) 125.946 (-1077.464 to 1329.357)	< 0.001 0.835	0.872
Body mass (kg)		**33.819 (16.663 to 50.976)**	**< 0.001**				
Age (years)		**-31.531 (-41.622 to -21.440)**	**< 0.001**	SRT (W) + age (years)	8.470 (7.341 to 9.599) -1.727 (-7.809 to 4.355)	< 0.001 0.572	0.873
BMI (kg/m^2^)		1.101 (-93.892 to 96.700)	0.977				
Body fat (kg)		**-80.440 (-110.870 to -50.011)**	**< 0.001**	SRT (W) + body fat (kg)	8.395 (7.363 to 9.427) -7.680 (-23.466 to 8.106)	< 0.001 0.334	0.874
FFM (kg)		**51.022 (34.242 to 67.803)**	**< 0.001**				
Hip-to-waist ratio		**2968.322 (453.721 to 5482.922)**	**0.021**				

*BMI* body mass index; *CI* confidence interval; *CPET* cardiopulmonary exercise testing; *SRT* steep ramp test; *V̇O*_*2peak*_ oxygen uptake at peak exercise; *WR*_*peak*_ work rate at peak exercise

## Discussion

This study aimed to investigate the test-retest reliability, as well as the criterion and construct validity of the SRT for assessing CRF in apparently healthy adults aged 25–85 years, and to develop an equation to predict V̇O_2peak_ from SRT performance. Results demonstrated excellent test-retest reliability and very high criterion validity of the SRT for assessing CRF in this population. Additionally, the hypotheses of strong correlations between SRT WR_peak_ and CPET V̇O₂_VT₁_, and between SRT WR_peak_ and CPET OUES, were confirmed, supporting the construct validity of the SRT as a measure of cardiorespiratory fitness. Moreover, SRT WR_peak_ can be used to predict V̇O_2peak_ measured during CPET, in which anthropometric variables do not contribute significantly to explaining its variation.

Our findings concerning the test-retest reliability of the SRT were comparable to earlier findings in healthy children and adolescents (ICC = 0.986) (Bongers et al. 2013), adults with COPD (ICC = 0.990) (Chura et al. 2012), adult cancer survivors (ICC = 0.996) (Backer et al. 2007), and males and females with type 2 diabetes (ICC = 0.951 and ICC = 0.908, respectively) (Rozenberg et al. 2015). The Bland-Altman plot showed that the mean difference in WR_peak_ between the first and second SRT was small and not clinically relevant. Some heteroscedasticity was observed in the absolute SRT WR_peak_ values (Fig. 1 graph A). The proportionality of the spread was confirmed by the only weak correlation between the mean values of both SRTs and their differences. The lack of heteroscedasticity in Fig. 1 graph B, in which the relative values are plotted against one another, further corroborates this observation. Among the lowest SRT values, observed in older adults, scores tended to fall below the zero line in the Bland-Altman plot (Fig. 1). In a larger sample of older adults, it should be investigated whether the SRT demonstrates lower stability, for instance as a result of a potential learning effect.

The relatively small SDC suggests that the SRT can detect improvements in CRF. However, evaluating the responsiveness of the SRT to detect changes in CRF over time remains an area for further research, which would provide valuable insights into the utility of the SRT for monitoring CRF.

Our findings regarding criterion validity are also comparable to earlier findings in healthy children and adolescents (*r* = 0.958) (Bongers et al. 2013), adult cancer survivors (*r* = 0.82; *r* = 0.86) (Backer et al. 2007; Weemaes et al^2021^), adults with type 2 diabetes (*r* = 0.90) (Rozenberg et al. 2015), adolescents with cystic fibrosis (*r* = 0.822) (Bongers et al. 2015), children with cancer (*r* = 0.883) (Braam et al. 2015), and adults with inflammatory bowel disease (*r* = 0.95) (Demers et al. 2024).

The developed equation enables the prediction of V̇O_2peak_ from SRT WR_peak_, explaining 87.4% of the variance in V̇O_2peak_ in healthy adults (SEE = 316 mL/min). Previous studies by De Backer et al. (2007) and Bongers et al. (2013) also developed prediction equations that explained 91.7% of the variance in V̇O_2peak_ in healthy children and adolescents (SEE = 237 mL/min) and 67.2% of the variance in V̇O_2peak_ in adult cancer survivors (SEE = 308 mL/min), respectively. At the group level, the equation developed in this study appears to be usable, as it explains a high proportion of the variance. At the individual level, however, the use of a prediction equation might result in substantial over- or underestimation of V̇O_2peak_. Therefore, future research should not only focus on the external validation of this prediction equation, but also on the development of sex- and age-specific norm values for SRT performance in adults, including older adults, which are currently lacking.

To the best of our knowledge, this study is the first to determine the correlation between SRT WR_peak_ and V̇O_2VT1_ as a submaximal CPET indicator of CRF, as well as the correlation between SRT WR_peak_ and the OUES as an effort-independent measure of CRF. Both the V̇O_2VT1_ and the OUES showed high correlations with SRT WR_peak_ in the current study. An earlier study by Balady et al. (2010), identified V̇O_2VT1_ as a submaximal indicator of CRF. Similarly, a scoping review by Akkerman et al. (2010) regarding the OUES, described high correlations between V̇O_2peak_ and V̇O_2VT1_, as well as between V̇O_2peak_ and OUES. Thus, the strong correlations found in the current study further support the construct validity of the SRT to assess CRF.

Benefits of the SRT compared to CPET as gold standard for assessing CRF are that it takes significantly less time (approximately 10 vs. 60 min) and does not require respiratory gas analyses. Accordingly, no specialized knowledge is needed to interpret its results. As such, the SRT can be considered to assess CRF in large populations (e.g., routine health checks). It is important to note that the SRT cannot replace CPET for diagnostic evaluation purposes. The SRT is primarily meant for a general evaluation of an individual’s CRF. In the case of suboptimal performance, a referral for CPET may be indicated. Another important consideration is that the accuracy of the SRT as an indicator of an individual’s CRF depends on the achievement of maximal effort (Trul-Kreuze et al. 2024). Since it is not possible to objectively assess whether maximal effort has been exerted during the SRT, it is crucial to provide clear pretest instructions, precise verbal guidance and consistent encouragement throughout the test, to ensure an accurate assessment of the individual’s CRF.

### Strengths and limitations

The strengths of this study include the broad age range of the participants in both the reliability and validity group, as well as the homogeneous distribution across these groups, and the fact that reporting was conducted according to the COSMIN guidelines. A limitation of this study is that the sequence of the tests was not randomized. Given the absence of statistically significant differences in resting heart rate values prior to the SRT and CPET, the potential influence of an order effect was considered negligible. Another limitation of this study is that the prediction equation has not yet been externally validated.

### Future research

To interpret SRT results in adults, including older adults, it is currently unavoidable to predict V̇O_2peak_ from SRT WR_peak_ for comparison with norm values. To enable direct comparison to norm values, future research should focus on expanding the existing norm values for SRT WR_peak_ in children, adolescents, and young adults with age- and sex-specific norm values for adults, including older adults. In the meantime, the existing equations to predict V̇O_2peak_ from SRT performance should be externally validated. Furthermore, evaluating the responsiveness of the SRT in detecting changes in CRF over time would provide valuable insights into its potential for monitoring longitudinal changes in an individual’s CRF. Finally, given the urgent need for practical measures to assess CRF in ageing populations, future research should investigate the applicability of the SRT in older patient populations, where comorbidities and disease characteristics may affect its validity, reliability, and safety.

## Conclusion

This study demonstrated excellent test-retest reliability, very high criterion validity, and high construct validity of the SRT for assessing CRF in apparently healthy adults, including older adults, suggesting that the SRT can be used to assess CRF in this population. A minimal detectable change in SRT WR_peak_ >15 W, > 0.2 W/kg or > 4.2% reflects a true change in an individual’s SRT performance.

## Supplementary Information

Below is the link to the electronic supplementary material.

Supplementary Material 1

## Data Availability

The datasets generated during and/or analyzed during the current study are available from the corresponding author on reasonable request.

## Abbreviations

6MWT: 6-minute walk test
BMI: Body mass index
CPET: Cardiopulmonary exercise testing
CRF: Cardiorespiratory fitness
ECG: Electrocardiography
EQ-6D: Six-dimensional EuroQol questionnaire
HR: Heart rate
HR_peak_: Heart rate at peak exercise
FFM: Fat-free mass
ICC: Intraclass correlation coefficient
IQR: Interquartile ranges
MEC-U: Medical research Ethics Committees United
MET: Metabolic equivalent
OUES: Oxygen uptake efficiency slop
PAR-Q: Physical activity readiness questionnaire
RER_peak_: Respiratory exchange ratio at peak exercise
SQUASH: Short questionnaire to assess health-enhancing physical activity
SDC: Smallest detectable change
SEE: Standard error of estimate
SEM: Standard error of the mean
SRT: Steep ramp test
V̇E: Minute ventilation
V̇E_peak_: Minute ventilation at peak exercise
V̇O_2_: Oxygen uptake
V̇O_2peak_: Oxygen uptake at peak exercise
V̇O_2VT1_: Oxygen uptake at the first ventilatory threshold
WR: Work rate
WR_peak_: Work rate at peak exercise
