# Supplementary material for: Reliability and validity of the steep ramp test to assess cardiorespiratory fitness in apparently healthy adults
Source: Eur J Appl Physiol. 2026 Jan 19;126(6):3115–27. doi: 10.1007/s00421-025-06071-y (PMC13287098; doi:10.1007/s00421-025-06071-y)
Supplement: Supplementary file 1 — Supplementary Material 1 [file 421_2025_6071_MOESM1_ESM.docx]

Supplemental Material 1. Specification of the equipment used at the different test locations.

| Materials | Test location | Manufacturer | Type | City | Country |
| --- | --- | --- | --- | --- | --- |
| Stadiometer (body height) | Hanze University of Applied Sciences | Seca | Seca 206 | Hamburg | Germany |
|  | Zuyd University of Applied Sciences;  Martini hospital Groningen | Seca | Seca 213 | Hamburg | Germany |
| Scale  (body mass) | Hanze University of Applied Sciences | Omron Healthcare Europe BV | Omron HN289 | Hoofddorp | The Netherlands |
|  | Zuyd University of Applied Sciences | Seca | Seca 760 | Hamburg | Germany |
|  | Martini hospital Groningen | Seca | Seca 799 | Hamburg | Germany |
| Measuring tape  (hip and waist circumference) | Hanze University of Applied Sciences; Zuyd University of Applied Sciences; Martini hospital Groningen | Seca | Seca 201 | Hamburg | Germany |
| Skinfold caliper  (skinfold thicknesses) | Hanze University of Applied Sciences; Zuyd University of Applied Sciences; Martini hospital Groningen | Baty International Ltd | Harpenden | Camberley | Britain |
| Cycle ergometer (SRT, CPET) | Hanze University of Applied Sciences; Martini hospital Groningen | Lode BV | Lode Corival Rehab | Groningen | The Netherlands |
|  | Zuyd University of Applied Sciences | Lode BV | Lode Excalibur | Groningen | The Netherlands |
| Cycle ergometer software (SRT) | Hanze University of Applied Sciences; Zuyd University of Applied Sciences; Martini hospital Groningen | Lode BV | Lode Ergometry Manager (LEM) Software | Groningen | The Netherlands |
| Heart rate monitor (heart rate) | Hanze University of Applied Sciences | Polar Elektro Oy | Polar T31 | Kempele | Finland |
|  | Zuyd University of Applied Sciences | Polar Elektro Oy | Polar H10 | Kempele | Finland |
|  | Martini hospital Groningen | Xand Cycling | Xand Cycling | - | China |
| Metabolic cart | Hanze University of Applied Sciences | Cortex GmbH | Meta Control 3000 | Leipzig | Germany |
| Abbreviations: CPET=cardiopulmonary exercise testing; SRT=steep ramp test. | | | | | |
